# Tumorous Stem Development of *Brassica Juncea*: A Complex Regulatory Network of Stem Formation and Identification of Key Genes in Glucosinolate Biosynthesis

**DOI:** 10.3390/plants9081006

**Published:** 2020-08-09

**Authors:** Mengyao Li, Fangjie Xie, Jie Li, Bo Sun, Ya Luo, Yong Zhang, Qing Chen, Yan Wang, Fen Zhang, Yunting Zhang, Yuanxiu Lin, Xiaorong Wang, Haoru Tang

**Affiliations:** 1 College of Horticulture, Sichuan Agricultural University, Chengdu 611130, China; limy@sicau.edu.cn (M.L.); xiefangjie@stu.sicau.edu.cn (F.X.); lijie@stu.sicau.edu.cn (J.L.); 14099@sicau.edu.cn (B.S.); 13621@sicau.edu.cn (Y.L.); Zhyong@sicau.edu.cn (Y.Z.); supnovel@sicau.edu.cn (Q.C.); wangyanwxy@sicau.edu.cn (Y.W.); zhangf@sicau.edu.cn (F.Z.); asyunting@sicau.edu.cn (Y.Z.); linyx@sicau.edu.cn (Y.L.); Wangxr@sicau.edu.cn (X.W.); 2 Institute of Pomology and Olericulture, Sichuan Agricultural University, Chengdu 611130, China

**Keywords:** *Brassica juncea*, transcriptome sequencing, differentially expressed genes, glucosinolates

## Abstract

Stem mustard is a stem variety of mustard, an important *Brassica* vegetable. The formation and development of the tumorous stem, which is the key organ for the direct yield and quality, is a complex biological process involving morphogenesis, material accumulation and gene regulation. In this study, we demonstrated through anatomical studies that stem swelling is mainly dependent on the increase in the number of cells and the volume of parenchyma cells in the cortex and pith. To further understand transcript and metabolic changes during stem swelling, we obtained 27,901 differentially expressed genes, of which 671 were specifically detected using transcriptome sequencing technology in all four stages of stem swelling. Functional annotation identified enrichment for genes involved in photosynthesis, energy metabolism, cell growth, sulfur metabolism and glucosinolate biosynthesis. Glucosinolates are a group of nitrogen- and sulfur-containing secondary metabolites, which largely exist in the Cruciferous vegetables. HPLC analysis of the contents and components of glucosinolates in four different stem development stages revealed eight glucosinolates, namely, three aliphatic glucosinolates (sinigrin, glucoalyssin and gluconapin), four indole glucosinolates (4-hydroxyglucobrassicin, glucobrassicin, 4-methoxyglucobrassicin and neoglucobrassicin) and one aromatic glucosinolate (gluconasturtiin). All these types of glucosinolates showed a significant downward trend during the stem swelling period. The content of aliphatic glucosinolates was the highest, with sinigrin being the main component. In addition, qPCR was used to validate the expression of nine genes involved in glucosinolate biosynthesis. Most of these genes were down-regulated during stem swelling in qPCR, which is consistent with transcriptome data. These data provide a basic resource for further molecular and genetic research on *Brassica juncea*.

## 1. Introduction

Stem mustard (*Brassica juncea* var. *tumida* Tsen et Lee) is an important vegetable crop that belongs to the genus *Brassica* in the Cruciferae family. In China, where it originated from, the tumorous stem of stem mustard provides the raw material for processing pickled products. Alongside the German pickled cabbage and European pickled cucumber [1], stem mustard pickles constitute the three most famous pickles globally. Since the development of a tumorous stem is directly related to the yield, quality and economic value of stem mustard, understanding the mechanism of stem swelling in stem mustard is important. The stem of the stem mustard has a unique morphological structure, with the petiole base forming 1–5 tumorous bulges by metamorphosis during the growth and development process and expanding laterally with the stem expansion (Figure S1) [2,3]. The tumor-like stem swelling of stem mustard is a complex physiological process, which involves various factors such as morphology, physiology, biochemistry and genetics. Based on the previous anatomical exploration of stem development mechanisms, an increase in the size and number of cells in the cortex and the medulla is the main cause of bulking [4]. Many studies investigating the molecular mechanism of the modified roots or stems development in vegetables such as potato, carrot, lotus and yam have been reported, wheares few studies exist on stem mustard. Endogenous hormones such as gibberellins and cytokinins and the photoperiod have been confirmed to regulate the formation of the swollen stem [5]. In addition, temperature and growth regulators could also affect the stem diameter of stem mustard [6]. Shi et al. [3] found that cell division and endoreduplication contribute to the stem swelling of stem mustard. Several GRAS transcription factor genes were highly expressed during stem swelling, implying their participation in the regulation of stem swelling [7]. Overexpression of *PHYA* in stem mustard has been shown to delay stem swelling [8]. Still, compared with other plants, there are very few reports on the discovery of genes related to stem swelling in stem mustard. Moreover, the molecular mechanism and regulatory network of the stem formation are yet to be elucidated.

Studies have shown the nutrients affecting the flavor quality of stem mustard to be proteins, amino acids, glucosinolates and saccharides. However, the flavor of mustard is mainly influenced by the hydrolysis products of glucosinolates [9]. Glucosinolates are secondary metabolites that contain nitrogen and sulfur and are widely found in cruciferous crops where they contribute to the unique flavor of cruciferous vegetables. Currently, over 120 kinds of glucosinolates have been identified [10]. Studies on glucosinolates in cruciferous vegetables such as mustard, Chinese cabbage, turnips and broccoli found that 2-propenyl glucosinolate and 2-hydroxy-3-butenyl glucosinolate contribute to the greatest influence on flavor [11]. Glucosinolates and their degradation products have a variety of biological activities in plant development and plant defense mechanisms [12]. In addition, some degradation products and derivatives of glucosinolates act as effective cancer-preventive agents in human health and nutrition [13,14]. There are significant differences in the types and contents of glucosinolates between species and varieties, while plant organs and development period also affect the content of glucosinolates [15,16]. Stem mustard is a widely eaten vegetable with a signature mustard flavor, and several studies have since been carried out to explore the composition and content of glucosinolates in stem mustard. A variance analysis indicated that the glucosinolates are primarily influenced by a species and tissue interaction [17]. Significant differences in glucosinolates were observed between different cultivars, with the highest content of glucosinolates being found in flower buds [18]. Although the swollen stem is used as the product organ of stem mustard, the pattern of change for glucosinolates in stem development is not clear. Therefore, understanding the change characteristics in the content and the biosynthetic pathway of glucosinolates in stem swelling of stem mustard could provide important theoretical significance and application prospects.

Despite several transcriptome reports for stem mustard, none of these studies have focused on cell structure or metabolite changes during development [7,19,20]. In this study, we aimed to further uncover differentially expressed genes (DEGs) and associated metabolic changes during stem swelling. Therefore, we performed transcriptome sequencing on the four stages of stem swelling of stem mustard and analyzed the data synthetically. By elucidating the dynamic changes in glucosinolate content and gene expression patterns during the period of stem development, our results could help reveal the biosynthesis pathway of glucosinolates in stem mustard.

## 2. Results

### 2.1. Anatomical Structures of Stem of B. Juncea

**Figure 1:**
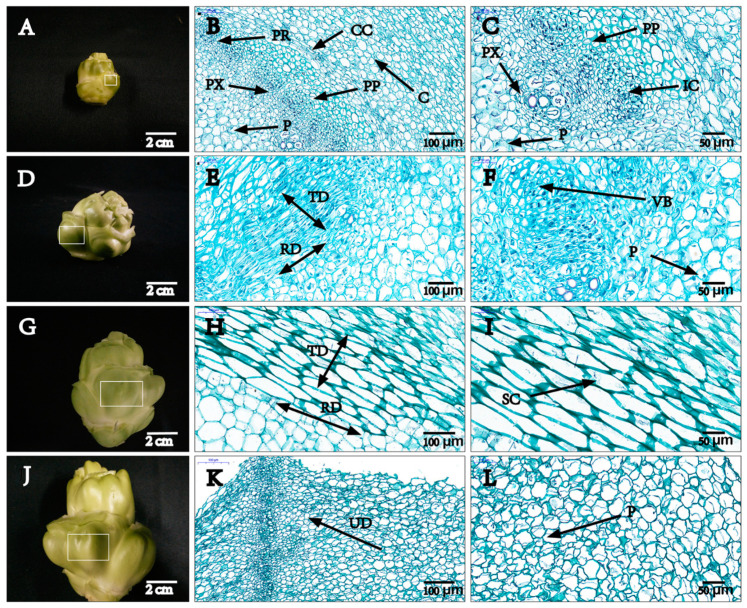
Stem anatomy at four different developmental stages (S1, S2, S3, S4). Scale bars: 2 cm (**A**,**D**,**G**,**J**); 100 μm (**B**,**E**,**H**,**K**); 50 μm (**C**,**F**,**I**,**L**). The transverse diameters are 2 cm in (A–C) (S1); 4 cm in (D–F) (S2); 6 cm in (G–I) (S3) and 8 cm in (J–L) (S4), respectively. I indicates tangential division cell layer, K indicates non-directional cell division area in the cortex and L indicates cell expansion. CC: Cork cambium. C: Cortex. IC: Intrafascicular cambium. P: Pith. PX: Primary xylem. PP: Primary phloem. PR: Pith ray. VB: Vascular bundle. TD: Tangential division. RD: Radial division. UD: Undirected division. SC: Spindle cells. The white box in **A**, **D**, **G**, **J** represents the site of observation.

To observe the expansion process, we focused on the cork cambium and pith. The expansion process involves tangential and radial mitosis of cambium parenchyma cells, which increases the number of cell layers, lengthens the stem and increases the diameter. The primary phloem forms the outer layer, with the new dividing cells arranged inside, while the primary xylem forms the inner layer, with the new dividing cells arranged outside. After parenchyma cells of the cambium have undergone mitosis, the swelling stem can grow in undirected division. With the continuous division of the cambium cells, the volume of medullary cells gradually increases as well as the formation of fleshy stems (Figure 1).

### 2.2. RNA-Seq Analysis

To investigate the transcriptome dynamics during stem swelling of stem mustard, we constructed and sequenced 12 cDNA libraries from four stages with three biological replicates using the Illumina HiSeq 4000 platform. A total of 574,333,958 raw reads were generated from 12 samples, and after filtering out adaptor sequences, ambiguous reads (reads with 5% unassigned (N) bases) and low-quality reads, 549,888,298 high-quality clean reads were obtained, which were further mapped to the *B*. *juncea* genome (V 1.5) using HISAT2 software. An average of 92.8% of the clean reads were mapped to the *B*. *juncea* genome and further used for reference-guided subsequent assembly and differential gene expression analysis. A summary of the Illumina sequencing data, reads assembly and reads mapped on the genome is shown in Table S1.

### 2.3. Global Gene Expression Analysis

**Figure 2:**
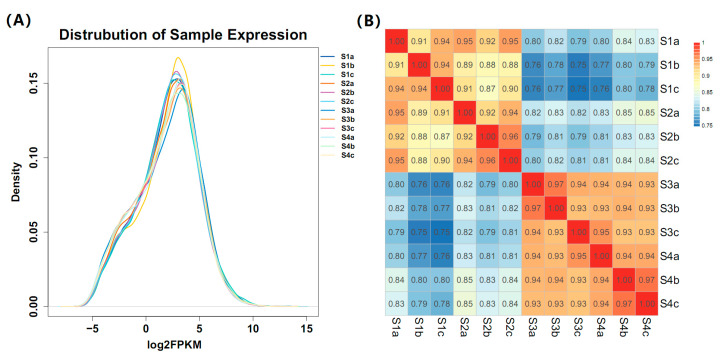
Overview of the gene expression profiles at four stem swelling stages in stem mustard. (**A**) The density of gene expression in each sample. The expression level of each gene was normalized as FPKM (fragments per kilobase of exon per million fragments mapped). FPKM values were log2-based. (**B**) Pearson’s correlation coefficients (PCCs) of gene expression among different stages. S1, S2, S3 and S4 indicate four stages, and a, b and c represent three biological replicates.

To obtain the global gene expression patterns during stem development, we normalized the expression levels of each gene as FPKM (fragments per kilobase of exon per million fragments mapped). A total of 59,174 transcripts were cumulatively expressed in all the samples. The density of gene expression (Log2^FPKM^) among all the samples exhibited similar expression profiles, indicating the reliability of biological sample collection and of the analytical method (Figure 2A). In addition, the correlation of the gene expression levels in any two samples was calculated to determine the parts of the transcriptome with similar functions/activities. As illustrated in Figure 2B, a higher correlation was observed in three biological replicates from the same stage. Moreover, the overall expression between pairs S1/S2 and S3/S4 was highly correlated compared with other stages, suggesting that the pairs could be closely related.

### 2.4. Differential Gene Expression

**Figure 3:**
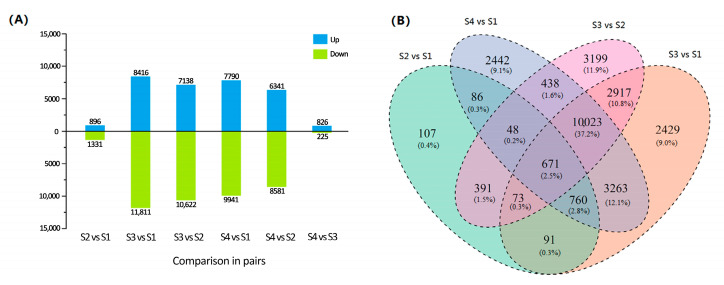
The distribution characteristics of differentially expressed genes (DEGs) in stem mustard during stem development. (**A**) The number of DEGs in any two different stages. The number of up- and down-regulated genes is presented via the bars above and below the *x*-axis, respectively. (**B**) Venn diagram of DEGs commonly and uniquely expressed among the comparison in pairs.

To investigate transcriptional differences in all the stem samples, we identified the DEGs using the threshold for false discovery rate (*q*-value) ≤ 0.05 and |log2 ratio| ≥ 1. A total of 27,901 genes in six comparison pairs were differentially expressed in at least one of the comparisons, at particular stages of stem swelling. The distribution information of DEGs in any two different stages is represented in Figure 3A and Tables S2–S7. Among the six comparisons, the number of genes ranged from 1051 (for S4 vs. S3) to 20,227 (for S3 vs. S1). The number of up-regulated DEGs was 896 and down-regulated DEGs was 1331 for S2 vs. S1. The up-regulated numbers of DEGs for S3 vs. S1, S3 vs. S2, S4 vs. S1 and S4 vs. S2 were 8416, 7138, 7790 and 6341, respectively. Moreover, the numbers of DEGs for S2 vs. S1 and S4 vs. S3 were markedly lower than others over the entire course of stem development. However, a marked fraction of specifically expressed genes was observed during S2 to S3. Except for the S4 vs. S3 comparison, a large number of genes were down-regulated compared with the up-regulated ones. This suggested that DEGs were apparent during stem development and, in particular, that an initial reorganization of gene expression was followed by the down-regulation of a great number of transcripts in later stages. Four comparison pairs (S2 vs. S1, S4 vs. S1, S3 vs. S2 and S3 vs. S1) were selected for the analysis of commonly and uniquely expressed genes in two or more comparisons (Figure 3B). Only 107 genes were exclusively present in S2 vs. S1, while 3199 genes were identified in S3 vs. S2. A significant proportion of DEGs (10,023 genes, accounting for 37.25%) were common in S4 vs. S1, S3 vs. S2 and S3 vs. S1. Of all DEGs, 671 genes were specifically detected in the four comparisons.

### 2.5. Dynamic Expression of DEGs in Stem Development

**Figure 4:**
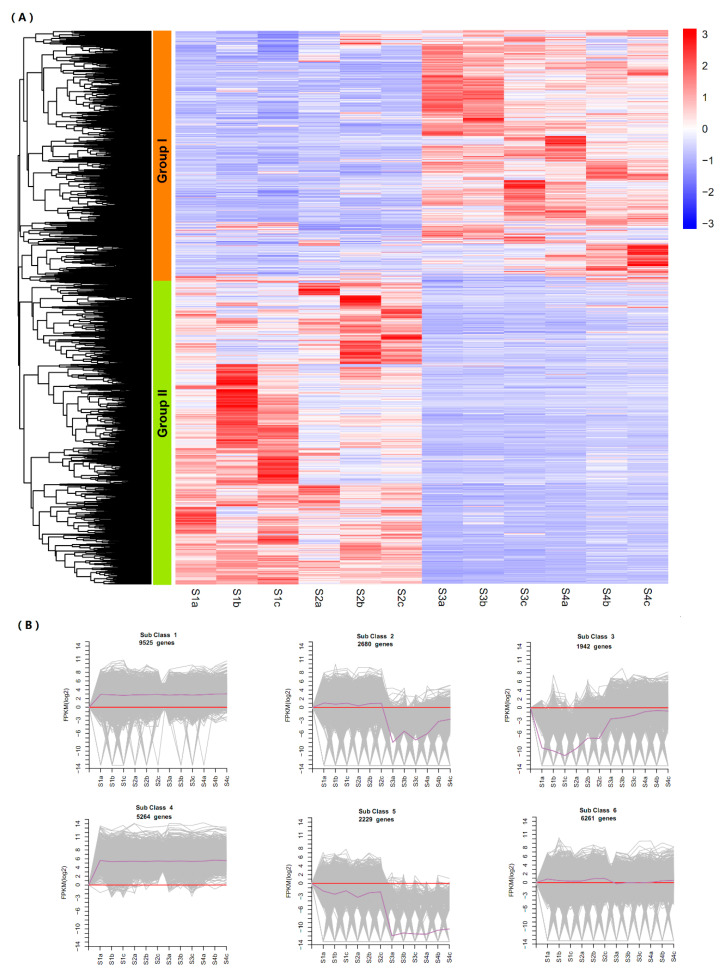
DEGs at four stem swelling stages in stem mustard. (**A**) Hierarchical clustering of DEGs for all samples. (**B**) K-mean clustering of gene expression trends. The expression profile of each gene in each cluster is shown as a gray line, and the average expression profile of all genes in each sample is shown as purple.

To gain a global view of expression patterns across four developmental stages, a heatmap of hierarchical clustering of the 27,901 DEGs was plotted using the normalized FPKM Z-score values (Figure 4A). All the DEGs were divided into two groups based on their expression patterns from S1 to S4. The genes in group I were highly expressed in S3 and S4, while group II members were highly expressed in S1 and S2. To further classify the expression patterns of all differential genes in each sample, we conducted a k-means clustering analysis (Figure 4B and Table S8) and identified six sub-classes with distinct average expressions. The expression level of 9,525 genes steadily increased between the four stages (Sub-Class 1). A total of 2680 genes were down-regulated between S3 and S4 stages (Sub-Class 2). There were 1942 genes decreased from S1 to S2, then markedly up-regulated at S3 (Sub-Class 3), while 5264 genes (Sub-Class 4) kept high expression during the whole development period. A total of 2229 genes changed little in S1 to S2 but markedly decreased from S2 to S3 (Sub-Class 5). In addition, 6261 genes showed no significant change trend (Sub-Class 6). Sub-Classes 1 and 4 contained genes that were predominantly expressed throughout the development period. The collection of genes in Sub-Classes 2 and 5 mostly showed relatively higher expression in S1 and S2, while genes in Sub-Class 3 exhibited higher expression in S3 and S4. In addition, it should be noted that most DEGs exhibited a significant change in the expression between S2 and S3 in the hierarchical and k-means clustering, suggesting that the difference in the transcriptional programs at S2 and S3 could determine developmental specificities during stem swelling.

### 2.6. Functional Analysis of DEGs

**Figure 5:**
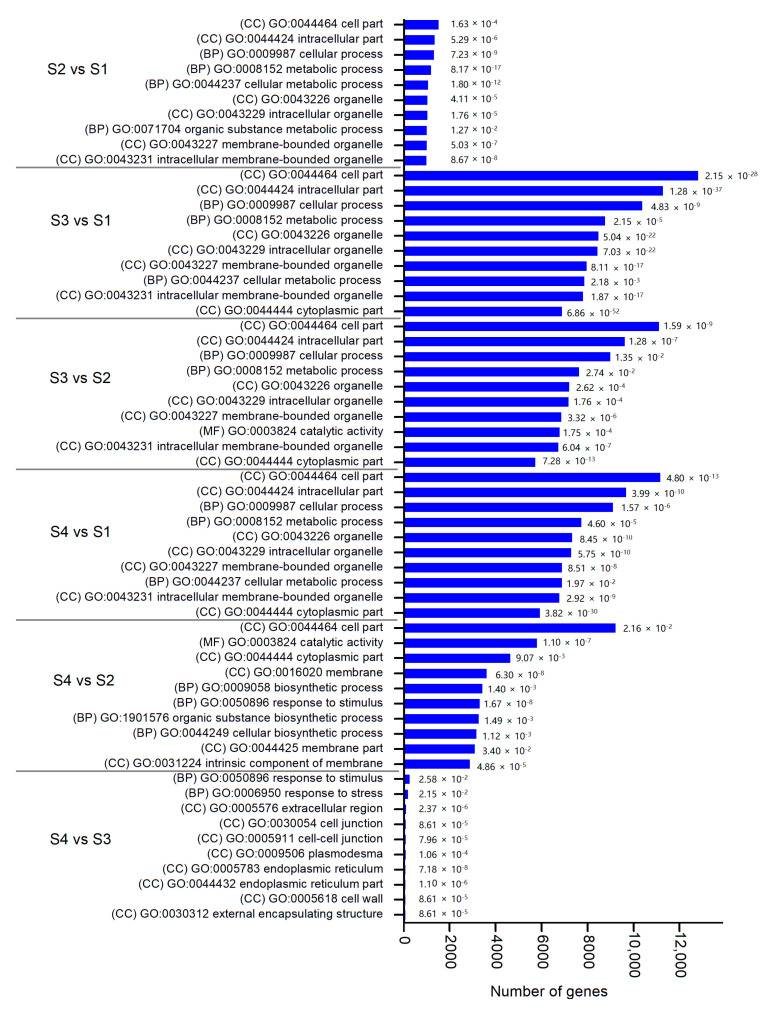
Gene Ontology (GO) enrichment analysis of DEGs among comparison pairs. The top 10 enriched terms with highly significant *p*-values (≤0.05) in each comparison are represented. The values of false discovery rate are shown in the bar graph. (BP): biological process; (MF): molecular function; (CC): cellular component.

Gene Ontology (GO) analysis was conducted to classify the annotation terms of DEGs for all the comparison pairs during stem swelling. The top 10 enriched terms belonging to the three main GO categories biological process (BP), cellular component (CC) and molecular function (MF) in each comparison are represented in Figure 5 and Table S9. Most DEGs were mainly enriched in cellular component, followed by biological process, suggesting that these genes may play a critical role in stem swelling. Genes associated with “cell part” and “intracellular part” were highly enriched in the cellular component except in S4 vs. S3, while “cellular process” and “metabolic process” were the main terms in the biological process. In addition, it is worth noting that more DEGs were down-regulated than up-regulated in all the comparison pairs except for S4 vs. S3. Of these, the largest number of down-regulated DEGs in S2 vs. S1, S3 vs. S1, S3 vs. S2 and S4 vs. S1 were significant enriched in “cell part”, followed by “intracellular part” and “cellular process”. A large number of genes were involved in cellular and metabolic processes, which revealed that cell division was more vigorous in early stages than later stages.

**Figure 6:**
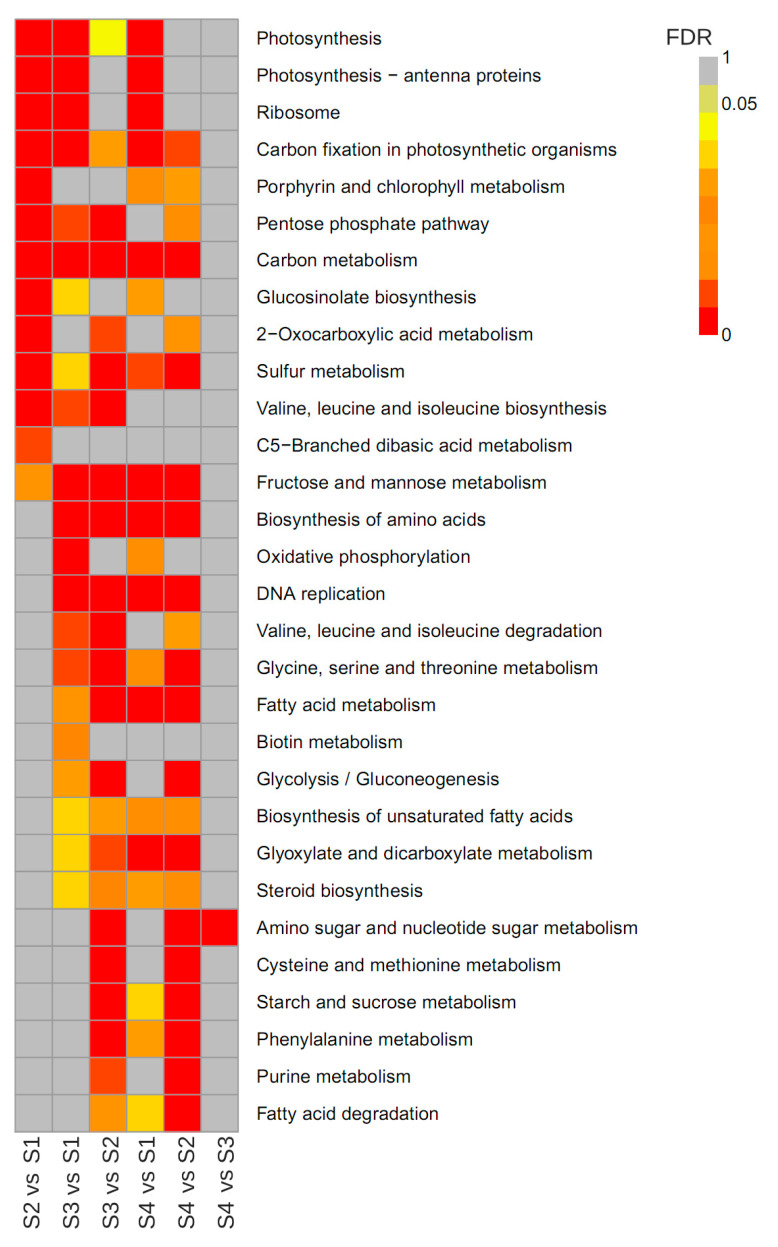
Kyoto Encyclopedia of Genes and Genomes (KEGG) enrichment analysis of DEGs was compared among comparison pairs. The map was made according to *q*-value of enrichment degree in the pathways, and the redder the color, the more significant the enrichment. The top 30 enriched pathways are represented. Significant DEGs were determined according to false discovery rate (FDR) ≤ 0.05 and (|log2 ratio| ≥ 1).

We further mapped the DEGs to the Kyoto Encyclopedia of Genes and Genomes (KEGG) database to analyze the metabolic pathways. Comparisons of the six pairs revealed that the enriched KEGG pathways varied substantially (Figure 6 and Table S10). More down-regulated genes were identified than up-regulated genes in all comparison pairs except for S4 vs. S3. In S1 comparison to other stages, terms such as “photosynthesis”, “photosynthesis-antenna proteins” and “ribosome” were significantly enriched. Most DEG genes related to “valine, leucine and isoleucine degradation” and “amino sugar and nucleotide sugar metabolism” were up-regulated than down-regulated in S3 vs. S2 and S4 vs. S2, suggesting that an important source sink transition occurred from S2 and S3, which contributes vigorous substance synthesis and storage material accumulation for cell division and volume increase. Additionally, several secondary metabolic pathways related to “carbon metabolism”, “glucosinolate biosynthesis” and “sulfur metabolism” were found to be significantly enriched.

### 2.7. Expression Characteristics of Genes Involved in Glucosinolate Biosynthesis

**Figure 7:**
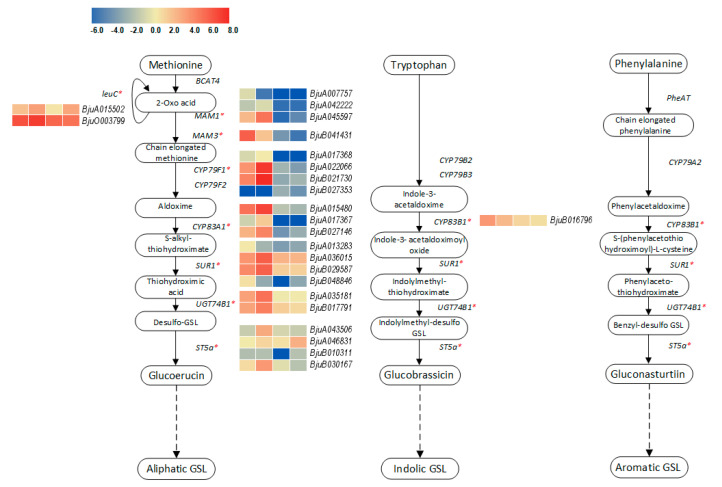
Schematic representation of genes associated with the core route of aliphatic, indole and aromatic glucosinolate biosynthesis in stem mustard. The expressions of DEGs in four stages are shown by the heatmap. The red asterisks indicate the DEGs (enzyme genes encoded by DEGs).

The glucosinolate biosynthesis pathway (ko00966) is the core pathway for the biosynthesis of plant bioactive glucosinolates from amino acids. Based on the different synthetic products, glucosinolate compounds can be biosynthesized via three routes, namely, the aliphatic, indole and aromatic glucosinolate pathways [10]. In this study, we identified a total of 66 genes belonging to 14 KEGG orthologies to be involved in the glucosinolate biosynthesis pathway (Table S11). Based on further differential expression analysis, 24 genes belonging to nine KEGG orthologies were differentially expressed in at least one comparison pair during the stem swelling (Figure 7 and Table S12). Except for S2 vs. S1, most genes were down-regulated in the other five comparison pairs. In addition, it was apparent that most of the genes expressed higher FPKM values in S1 and S2, and lower FPKM values in S3 and S4. However, genes encoding enzymes such as methionine transaminase (BCAT4), homomethionine N-monooxygenase (CYP79F2), tryptophan N-monooxygenase (CYP79B2, CYP79B3), PheAT, and phenylalanine N-monooxygenase (CYP79A2) did not have any significant difference in expression.

### 2.8. Validation of the Expression of DEGs by qPCR

**Figure 8:**
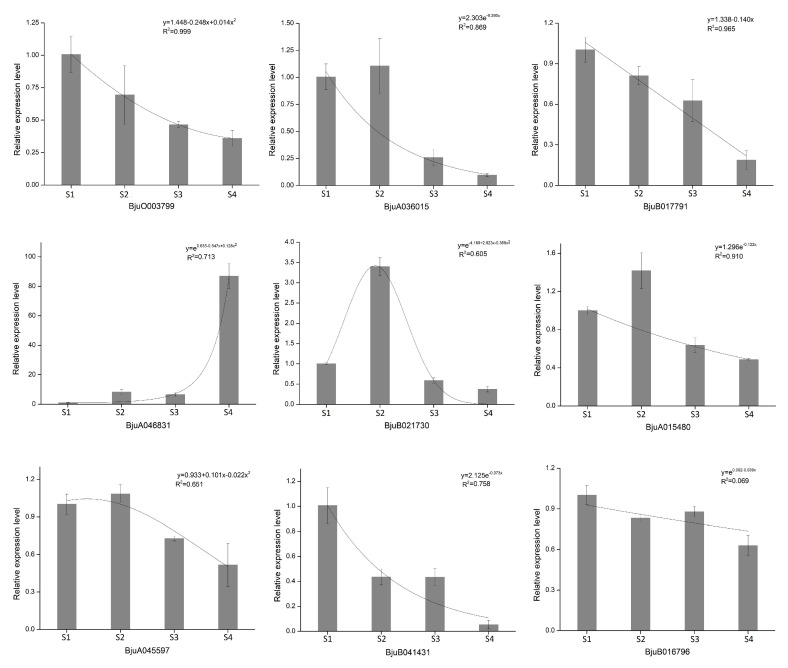
qPCR analysis of DEGs involved in glucosinolate biosynthesis in stem mustard.

To evaluate the validity of the transcript abundance obtained by RNA-seq, a total of nine DEGs derived from the glucosinolate biosynthetic pathway were selected for qPCR analysis. As shown in Figure 8, most of these genes were down-regulated during stem swelling. Overall, we found that the qPCR and RNA-Seq results were comparable (compare Figure 8 with the heatmap in Figure 7). The expression of *BjuA036015*, *BjuA015480* and *BjuA045597* was higher in S1 and S2 than in S3 and S4, whereas *BjuA046831* was markedly up-regulated in S3 and S4. Furthermore, the expression level of *BjuB021730* was gradually increased from S1 and peaked in S2, then decreased. Regression analysis performed to characterize the association between the development stage and expression level further demonstrated that, unlike *BjuB016796*, the other eight genes had a high degree of fit between them as revealed by the coefficient of determination (*R*^2^).

### 2.9. Determination of the Contents and Distribution of Glucosinolates

**Figure 9:**
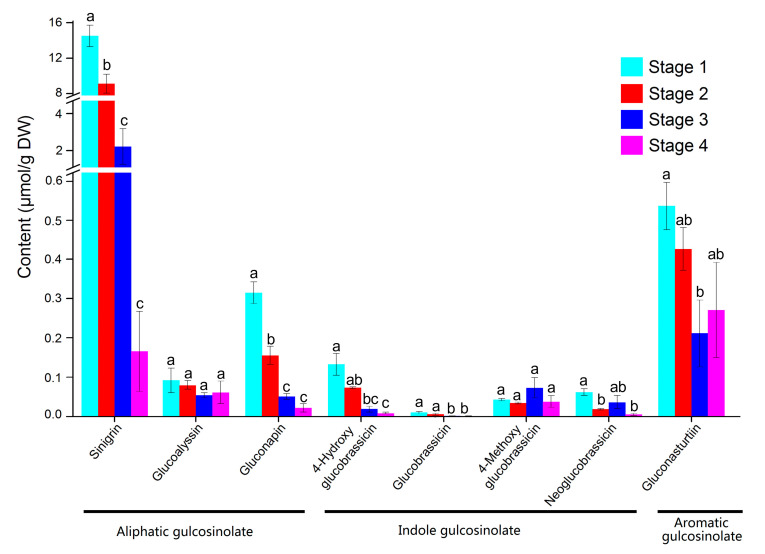
The composition and content of glucosinolates (GLSs) in different stages in stem mustard. For each tissue, the same letter in the same testing method means no significant differences (*p* < 0.05) according to the Least Significant Differences (LSD) test and Tukey tests.

To directly connect glucosinolate biosynthesis genes with the glucosinolate compounds during stem swelling, the contents and distribution of glucosinolates in different stages in stem mustard were examined by HPLC. Eight glucosinolates were identified in four-stage samples, namely, three aliphatic glucosinolates (sinigrin, glucoalyssin and gluconapin), four indole glucosinolates (4-hydroxyglucobrassicin, glucobrassicin, 4-methoxyglucobrassicin and neoglucobrassicin), and one aromatic glucosinolate (gluconasturtiin) (Figure 9 and Table S13).

The contents of total glucosinolates were significantly different in the four stages. S1 had the highest content of total glucosinolates, which was about 1.6 times, 5.9 times and 27.4 times higher than S2, S3 and S4, respectively. In S1, aliphatic glucosinolates accounted for 94.98% of the total glucosinolates, followed by aromatic and indole glucosinolates. Among them, sinigrin was the major component with a ratio of 92.39%, while the other seven types of glucosinolates accounted for only 7.61%. The distribution trends of glucosinolates in S2 and S3 were similar to that in S1. Moreover, in the aliphatic glucosinolates, the content of each component decreased with the increase in stem expansion stage, which was highly presented in S2 and S3, at 94.38% and 87.22% of the total glucosinolates, respectively. In S4, the total content of glucosinolates was relatively low and mainly comprised gluconasturtiin and sinigrin. In indole glucosinolates, the contents of 4-hydroxy gucobassicin and gucobassicin decreased with the increase in stem expansion period; 4-methoxy glucobrassicin and neoglucobrassicin first decreased, then increased and then decreased, and the contents of S2 and S4 were the lowest. In aromatic glucosinolates, gluconasturtiin content at S1 was the highest, while that at S3 was the lowest. Besides, the correlation analysis showed that the content of the eight glucosinolates presented a negative correlation with the development of the stem (Table S14), while the constituents of glucosinolates presented a positive correlation. In particular, the content of sinigrin showed a highly positive correlation with gluconapin, 4-hydroxy glucobrassicin, glucobrassicin and gluconasturtiin.

## 3. Discussion

### 3.1. Transcriptome Sequencing Depicts Dynamic Changes in Gene Expression and Metabolism During Stem Swelling in B. Juncea

Stem mustard is an important economic crop, cultivated for its tumor stem, which determines its yield and quality. The focus of most studies has been on the physiology and biochemistry of different varieties of stem mustard. By contrast, knowledge of genes and metabolic pathways controlling stem development is limited. RNA-seq is a powerful technique that allows the identification of new genes and large-scale analyses of gene expression [21,22]. Additionally, RNA-seq has been successfully used to study certain organs. For example, the single cell-type root hair transcriptome was less complex than the transcriptome of multiple cell-type primary roots without root hairs for maize [23]. mRNA and miRNA sequencing data also uncovered a regulatory network of tuber expansion in yam [24]. Moreover, the transcriptome sequencing technology provides a more comprehensive characterization and quantitative description compared with the gene chip technology [25].

In this study, we discovered that the stem swelling of stem mustard was mainly attributed to the increase in the number of cells in the cortex and pith and the volume of parenchyma cells. The cambium cells of the tumor stem first underwent tangential division during the S2 and S3 periods to increase the number of cell layers, which were then divided by radial division to form the long axis of the stem. In particular, during the S3 period, the cell volume is significantly increased, and the uplift is more obvious. From S2 to S3, undirected division accompanied by cell enlargement occurred in the tissues; this result was in line with the finding of Kolomiets [26]. Anatomical studies of potato indicated that the cortical and pith cells are involved in enlargement and constitute a large proportion of mature tubers [26,27]. Previous studies have elucidated the heavy involvement of genes in the processes of cell division and cell expansion. For instance, *CycD3* expression was found to correlate with fleshy roots [28]. The cell wall regulatory genes *BjXTH1* and *BjXTH2* were specifically expressed in the medullary cell for *B. juncea* [3,29]. In this study, a large number of DEGs were identified through transcriptome analysis. Based on the GO categories, we revealed that many of the DEGs participated in “metabolic process” and “cellular process”, especially in S2 and S3 stages, an indication that these genes are involved in important cell regulatory processes such as cell growth, cell development and cell differentiation. A related founding in morphological analysis is that the stem grew rapidly at the S2 and S3 stages and the cell division was vigorous. The photosynthate provides the material base for the growth of plant organs [30,31]. In this study, KEGG pathways significantly enriched included photosynthesis, antenna protein, carbon metabolism and the ribosome in the early stage of stem swelling. However, the pathways of “starch and sucrose metabolism”, “glycolysis/gluconeogenesis” and “amino acid transport and metabolism” were enriched in the later stages of expansion. This could be due to the changes in materials and energy metabolism in stem development. In the early stage of stem swelling, plants often allocate more biomass to aboveground organs to achieve high leaf photosynthesis, suggesting that these genes play a key role in the early stage of development. Thus, the stem can be a powerful metabolic pool, which is dominated by the photosynthates. However, since the stem showed a state of reduced basic metabolic activity in the early stages, we speculated that it would act as a storage bank of carbohydrates and proteins at a late stage in development. Based on previous findings, most of the genes identified in this study are functionally involved in metabolic processes, especially in the regulatory processes of starch and sucrose metabolism, as well as storage material accumulation to provide materials and energy in the process of organ development [32,33].

### 3.2. Identification of Key Genes and Diversity in Glucosinolate Biosynthesis

Glucosinolates are a group of nitrogen- and sulfur-containing secondary metabolites that are derived from glucose and amino acids. Along with their derivatives, glucosinolates play an important role in the growth and development of cruciferous plants, plant defense, food flavor, anti-cancer effects etc. [34,35]. Extensive studies have revealed that glucosinolate is intimately linked to carbon and nitrogen metabolism. Marino et al. [36] indicated that *Arabidopsis* showed accumulation of glucosinolate content and induction of myrosinase activity under ammonium nutrition. Overall, the availability and assimilation of sulfur were positively correlated with the level of aliphatic glucosinolates, while the availability of nitrogen was positively correlated with the level of indole glucosinolates [37,38]. The biosynthesis pathway of glucosinolates mainly consists of three steps, namely, the extension of the side chain of the precursor amino acid, the formation of the core structure and the secondary modification, involving multiple gene families such as BCAT, MAM, CYP79 and CYP83 of CYP450, and SUR1 [39,40]. In this study, most of the genes involved in the sulfur metabolism and glucosinolate biosynthesis were enriched, with more up-regulated genes than down-regulated genes in S1 and S2 stages. The content of glucosinolates was highest in S1 and S2, indicating that these genes may determine the synthesis of glucosinolates. We identified 66 genes involved in the biosynthesis of glucosinolates and used qPCR to validate some of the highly DEGs. Most of the genes were highly expressed at the early stages and lower at the later stages. Some genes such as *BjuA036015* (belonging to SUR1), *BjuA045597* (MAM1), *BjuB021730* (CYP79F1) and *BjuA015480* (CYP83A1) initially increased and then declined during stem development. The expression levels of *BjuB041431* (MAM3) and *BjuB016796* (CYP83B1) genes were highest at S1. Our comparisons of glucosinolate content in the tumor stem at different swelling stages revealed that the content of glucosinolates declined during the swelling stage, which was consistent with the qPCR verification results. Moreover, previous studies have shown that silencing the *MAM* gene can significantly induce the production of 2-propenyl glucosinolate in *Brassica napus* [41], and overexpression of the *SUR1* gene in *Arabidopsis* can increase the content of aliphatic glucosinolates and indole glucosinolates [42]. The above-mentioned studies indicate that these genes may play an important regulatory role in promoting glucosinolate synthesis in the early stage of stem swelling.

In addition, glucosinolates are diverse in different plant species. For instance, turnip glucosinolates mainly comprise 3-butenyl glucosinolate and indole-3-methyl glucosinolate [43], while the Korean Chinese cabbage is dominated by 3-butenyl glucosinolates, 4-pentyl glucosinolates and 2-hydroxy-3-butenyl glucosinolates [44]. Unlike most of the Chinese cabbages, which contain a lower amount of aliphatic than indolic glucosinolates, broccoli, turnip and rapeseed all have much higher aliphatic glucosinolate content compared with indolic glucosinolate content [45]. Moreover, previous studies identified six, eight and four major glucosinolates in broccolini, broccoli and Chinese broccoli seeds, respectively [46]. Glucosinolate content also differs in different varieties of the same species. For instance, D Manh et al. [47] found the content of indole glucosinolate in mustard to be higher than that in red mustard hairy roots, but the content of aromatic glucosinolate in red mustard was significantly higher than that in green mustard. Although Thomas et al. [48] showed that the highest content in mustard was 2-propenyl glucosinolate, He et al. [49] could not detect this glucosinolate. In this study, the quantitative analysis of glucosinolates in the tumorous stem of *B. juncea* showed that the content of aliphatic glucosinolates was the highest, accounting for more than 90% of the total glucosinolates, which was consistent with the findings of Wang et al. [50] and Augustine et al. [51]. Similarly, the quantity and composition of glucosinolates changes dynamically with the age of the plants, the studied tissues and environmental conditions [52,53].

This study provides a comprehensive analysis of global changes in gene expression during stem swelling in mustard. qPCR analysis of glucosinolate production showed decreasing expression of biosynthetic genes and glucosinolate content in swelling stems. These findings provide a foundation for further genetic and molecular research on stem mustard.

## 4. Materials and Methods

### 4.1. Plant Materials

The stem mustard (*Brassica juncea* var. *tumida* Tsen et Lee) cultivar ‘Fuza No. 2’ was used in this study. The plants were grown in the field in Chongzhou practice base, Sichuan Province of China. Digital calipers were used to measure the stem from different angles and to take the maximum diameter precisely. Swelling stems were collected in three biological replicates with stem diameters at 2 cm (plants grown for 15 weeks), 4 cm (plants grown for 17 weeks), 6 cm (plants grown for 19 weeks) and 8 cm (plants grown for 20 weeks), representing S1, S2, S3 and S4 stages, respectively (Figure 1). The tissue samples were immediately frozen in liquid nitrogen and then stored at −80 °C for RNA-seq and qPCR.

### 4.2. Anatomy Study on Stem Swelling of Stem Mustard

The stem swelling was divided into four stages based on plants’ development. Stems were added to 8 mL of 50% FAA fixative solution (V formalin: V glacial acetic acid: V 50% alcohol = 1:1:18) and fixed at 4 °C for 24 h, dehydrated with ethanol and then embedded in paraffin. The thickness of the slice was 1 cm^3^, and safranin O-fast green reagent was used to stain stem tissues. Morphological structure at each developmental stage was observed by CaseViewer and photographed.

### 4.3. RNA Isolation and cDNA Synthesis, Sequencing and de Novo Assembly

Total RNA was extracted using the total RNA kit (Tiangen, Beijing, China). The quantity and quality of RNA samples were measured by agarose gel electrophoresis and the use of a Nanodrop ND 1000 spectrophotometer (Nanodrop Technologies Inc., Delaware, DE, USA). Only the samples with an A260/A280 ratio of 1.8–2.2 and an A260/A230 ratio > 1.8 were used for further analysis. Total RNA (1.0 µg) was reverse-transcribed into cDNA using a PrimeScript RT reagent kit (Takara, Dalian, China). RNaseH and dNTPs were added to synthesize the second strand of cDNA in the DNA polymerase I system. The purified double-stranded cDNA was amplified by PCR, the products were further purified to obtain libraries for Illumina HiSeq 4000 sequencing, and 150 bp paired-end reads were generated (Novogene Technologies Co., Ltd., Beijing, China). The transcriptome data were submitted to NCBI Sequence Read Archive (http://trace.ncbi.nlm.nih.gov/Traces/sra/) under the accession number SRP151320. The genome sequences of *B. juncea* genome (V 1.5) were downloaded from the *Brassica* database (http://brassicadb.org/brad/).

### 4.4. Differential Gene Analysis

Transcript abundance for all unigenes in each sample was counted by HTSeq, and FPKM (fragments per kilo base of exon per million fragments mapped) was then calculated to estimate the expression level. DESeq2 was designed for differential gene expression analysis between two samples with biological replicates [54]; genes with false discovery rate (*q*-value) ≤ 0.05 and |log2_ratio| ≥ 1 are identified as DEGs. Hierarchical and k-means clustering of gene expression trends was applied to the DEGs by heatmap [55].

### 4.5. Function Annotation

The Gene Ontology (GO) enrichment of DEGs was implemented by the hypergeometric test to exhibit the biological functions of the DEGs. In addition, the Kyoto Encyclopedia of Genes and Genomes (KEGG) was used to analyze the metabolic pathways. Both the GO terms and KEGG terms with *p*-value ≤ 0.05 were considered to be significantly enriched.

### 4.6. Quantitative Real Time PCR (qPCR) Analysis

qPCR was performed using the Bio-Rad CFX96TM real-time PCR System (Bio-Rad, Hercules, CA, USA) with the 2 × T5 Fast qPCR Mix (SYBR GreenI) (TsingKe, Beijing, China). The gene-specific primers were designed by Primer Premier 6, and the primer sequences are shown in Table S15. Each 20 µL PCR reaction contained 2.0 µL of diluted cDNA, 0.4 µL of each primer (10 mM), 10 µL of SYBR Green I mix (Takara, Dalian, China), and 7.2 µL of ddH_2_O. The PCR conditions were as follows: at 95 °C for 1 min for pre-denaturation, 40 cycles of 95 °C for 10 s for denaturation, and 58 °C for 15 s for annealing and extension. A melting curve (65–95 °C, at increments of 0.5 °C) was generated to verify the specificity of primer amplification. All experiments were performed with three biological replicates and three technical replicates for each tissue sample. The normalization of relative expression level of each gene was done with the reference gene *UBC* [29], and the fold change was calculated using the 2^−ΔΔCT^ method [56].

### 4.7. Glucosinolate Compound Analysis

The glucosinolates were extracted and analyzed as described by Sun et al. [17]. Briefly, the freeze-dried samples of four stages were boiled for 20 min in 1 mL of distilled water; the supernatant was collected after centrifugation (7000× *g*, 5 min), and the extraction step repeated once; the supernatants were mixed before being applied onto an analytical column using a DEAE-Sephadex A-25 (40 mg) column (pyridine acetate form) (GE Healthcare, Piscataway, NJ, USA). The glucosinolates were converted into their desulpho analogs after treatment with 200 μL of 0.1% aryl sulphatase for 16 h at room temperature. The resulting desulpho glucosinolates were then eluted with two washes (0.5 mL) of water, and the eluates were mixed and analyzed using a Waters HPLC instrument equipped with a model 2996 PDA absorbance detector (Waters, Milford, MA, USA). The samples were separated on a Waters Symmetry C18 column (250 × 4.6 mm i.d.; 5 μm) and data obtained with the absorbance at 226 nm. Ortho-nitrophenyl-β-d-galactopyranoside was used as an internal standard, and the normalization of peak-areas was used to calculate the glucosinolates.

## Supplementary Materials

The following are available online at https://www.mdpi.com/2223-7747/9/8/1006/s1, Figure S1: Figure S1. The adult plant of stem mustard, Table S1: Sequencing statistics of the transcriptome from four development stages of swelling stems, Table S2: A dataset of significantly up and down-regulated DEGs in S2 vs. S1, Table S3: A dataset of significantly up and down-regulated DEGs in S3 vs. S1, Table S4: A dataset of significantly up and down-regulated DEGs in S3 vs. S2, Table S5: A dataset of significantly up and down-regulated DEGs in S4 vs. S1, Table S6: A dataset of significantly up and down-regulated DEGs in S4 vs. S2, Table S7: A dataset of significantly up and down-regulated DEGs in S4 vs. S3, Table S8: The expression profiles of all individual genes assigned to each cluster, Table S9: GO enrichment analysis of DEGs among comparison pairs, Table S10: KEGG enrichment analysis of DEGs was compared among comparison pairs, Table S11: Associated genes in the glucosinolate biosynthetic pathway, Table S12: Differentially expressed genes in the glucosinolate biosynthetic pathway, Table S13: The contents and distribution of glucosinolates in different stages in stem mustard, Table S14: The correlation between the glucosinolates compounds and development stage was expressed, Table S15: Sequences of primers used in qPCR.
