# Supplementary material for: Tumorous Stem Development of Brassica Juncea: A Complex Regulatory Network of Stem Formation and Identification of Key Genes in Glucosinolate Biosynthesis
Source: Plants (Basel). 2020 Aug 9;9(8):1006. doi: 10.3390/plants9081006 (PMC7466272; doi:10.3390/plants9081006)
Supplement: Supplementary file 1 [file plants-09-01006-s001.zip › Supplementary File-Proof/Figure S1.doc]

**Figure S1.** The adult plant of stem mustard. (**A**) Whole plant of stem mustard; (**B**) The swollen stem of stem mustard.
