# Supplementary material for: Tumorous Stem Development of Brassica Juncea: A Complex Regulatory Network of Stem Formation and Identification of Key Genes in Glucosinolate Biosynthesis
Source: Plants (Basel). 2020 Aug 9;9(8):1006. doi: 10.3390/plants9081006 (PMC7466272; doi:10.3390/plants9081006)
Supplement: Supplementary file 1 [file plants-09-01006-s001.zip › Supplementary File-Proof/Table S1 and S9-S15.doc]

**Table S1.** Sequencing statistics of the transcriptome from four development stages of swelling stems.

|  | **S1a** | **S1b** | **S1c** | **S2a** | **S2b** | **S2c** | **S3a** | **S3b** | **S3c** | **S4a** | **S4b** | **S4c** |
| --- | --- | --- | --- | --- | --- | --- | --- | --- | --- | --- | --- | --- |
| Raw Reads Number | 46,965,010 | 48,874,792 | 46,814,520 | 47,488,074 | 47,269,242 | 48,908,880 | 47,536,890 | 48,329,204 | 47,750,250 | 49,238,016 | 46,647,784 | 48,511,296 |
| Raw Bases Number | 7,044,751,500 | 7,331,218,800 | 7,022,178,000 | 7,123,211,100 | 7,090,386,300 | 7,336,332,000 | 7,130,533,500 | 7,249,380,600 | 7,162,537,500 | 7,385,702,400 | 6,997,167,600 | 7,276,694,400 |
| Clean Reads Number | 44,934,678 | 46,944,272 | 45,051,076 | 45,782,994 | 45,236,824 | 46,679,284 | 44,294,060 | 46,681,102 | 46,265,490 | 47,456,690 | 44,516,656 | 46,045,172 |
| Clean Reads Rate(%) | 95.68 | 96.05 | 96.23 | 96.41 | 95.7 | 95.44 | 93.18 | 96.59 | 96.89 | 96.38 | 95.43 | 94.92 |
| Clean Bases Number | 6,740,201,700 | 7,041,640,800 | 6,757,661,400 | 6,867,449,100 | 6,785,523,600 | 7,001,892,600 | 6,644,109,000 | 7,002,165,300 | 6,939,823,500 | 7,118,503,500 | 6,677,498,400 | 6,906,775,800 |
| Low-quality Reads Number | 240,458 | 340,108 | 358,360 | 335,618 | 332,888 | 433,408 | 314,306 | 418,582 | 352,612 | 348,260 | 331,188 | 349,318 |
| Low-quality Reads Rate(%) | 0.51 | 0.7 | 0.77 | 0.71 | 0.7 | 0.89 | 0.66 | 0.87 | 0.74 | 0.71 | 0.71 | 0.72 |
| Ns Reads Number | 17,574 | 18,028 | 17,960 | 17,408 | 18,660 | 18,554 | 17,982 | 19,346 | 18,384 | 18,878 | 18,940 | 15,856 |
| Ns Reads Rate(%) | 0.04 | 0.04 | 0.04 | 0.04 | 0.04 | 0.04 | 0.04 | 0.04 | 0.04 | 0.04 | 0.04 | 0.03 |
| Adapter Polluted Reads Number | 1,772,300 | 1,572,384 | 1,387,124 | 1,352,054 | 1,680,870 | 1,777,634 | 2,910,542 | 1,210,174 | 1,113,764 | 1,414,188 | 1,781,000 | 2,100,950 |
| Adapter Polluted Reads Rate(%) | 3.77 | 3.22 | 2.96 | 2.85 | 3.56 | 3.63 | 6.12 | 2.5 | 2.33 | 2.87 | 3.82 | 4.33 |
| Raw Q30 Bases Rate(%) | 96.46 | 96 | 95.96 | 96.01 | 96.02 | 95.61 | 96.11 | 95.7 | 95.87 | 95.91 | 95.96 | 96.18 |
| Clean Q30 Bases Rate(%) | 96.69 | 96.32 | 96.34 | 96.36 | 96.34 | 96.01 | 96.38 | 96.14 | 96.24 | 96.25 | 96.3 | 96.51 |
| Total Gene Number | 49,562 | 48,654 | 48,165 | 48,335 | 50,407 | 50,517 | 45,965 | 47,403 | 46,824 | 47,408 | 48,978 | 49,394 |
| Mapped Reads | 41,909,035 | 43,806,651 | 41,958,666 | 42,650,468 | 42,294,070 | 43,485,531 | 41,292,866 | 43,417,577 | 41,233,559 | 44,124,525 | 41,632,533 | 42,948,403 |
| MappingRate to the Genome | 0.9327 | 0.9332 | 0.9314 | 0.9316 | 0.9349 | 0.9316 | 0.9322 | 0.9301 | 0.8912 | 0.9298 | 0.9352 | 0.9327 |

Each stage has three biological replicates.

**Table S9. GO enrichment analysis of DEGs among comparison pairs.**

| **Comparison in Pairs** | **GO Categories** | **Term Name** | **Accession** | **FDR** | **Total DEG** | **Up** | **Down** |
| --- | --- | --- | --- | --- | --- | --- | --- |
| S2 vs S1 | CC | cell part | GO:0044464 | 1.63E-04 | 1509 | 609 | 900 |
|  | CC | intracellular part | GO:0044424 | 5.29E-06 | 1336 | 504 | 832 |
|  | BP | cellular process | GO:0009987 | 7.23E-09 | 1298 | 514 | 784 |
|  | BP | metabolic process | GO:0008152 | 8.17E-17 | 1175 | 449 | 726 |
|  | BP | cellular metabolic process | GO:0044237 | 1.80E-12 | 1050 | 403 | 647 |
|  | CC | organelle | GO:0043226 | 4.11E-05 | 1021 | 365 | 656 |
|  | CC | intracellular organelle | GO:0043229 | 1.76E-05 | 1019 | 364 | 655 |
|  | BP | organic substance metabolic process | GO:0071704 | 1.27E-02 | 990 | 409 | 581 |
|  | CC | membrane-bounded organelle | GO:0043227 | 5.03E-07 | 987 | 357 | 630 |
|  | CC | intracellular membrane-bounded organelle | GO:0043231 | 8.67E-08 | 975 | 347 | 628 |
| S3 vs S1 | CC | cell part | GO:0044464 | 2.15E-28 | 12,792 | 5089 | 7703 |
|  | CC | intracellular part | GO:0044424 | 1.28E-37 | 11,255 | 4532 | 6723 |
|  | BP | cellular process | GO:0009987 | 4.83E-09 | 10,358 | 4073 | 6285 |
|  | BP | metabolic process | GO:0008152 | 2.15E-05 | 8747 | 3445 | 5302 |
|  | CC | organelle | GO:0043226 | 5.04E-22 | 8460 | 3392 | 5068 |
|  | CC | intracellular organelle | GO:0043229 | 7.03E-22 | 8408 | 3365 | 5043 |
|  | CC | membrane-bounded organelle | GO:0043227 | 8.11E-17 | 7937 | 3236 | 4701 |
|  | BP | cellular metabolic process | GO:0044237 | 0.002179 | 7836 | 3119 | 4717 |
|  | CC | intracellular membrane-bounded organelle | GO:0043231 | 1.87E-17 | 7786 | 3148 | 4638 |
|  | CC | cytoplasmic part | GO:0044444 | 6.86E-52 | 6863 | 2374 | 4489 |
| S3 vs S2 | CC | cell part | GO:0044464 | 1.59E-09 | 11,087 | 4197 | 6890 |
|  | CC | intracellular part | GO:0044424 | 1.28E-07 | 9604 | 3768 | 5836 |
|  | BP | cellular process | GO:0009987 | 1.35E-02 | 8974 | 3361 | 5613 |
|  | BP | metabolic process | GO:0008152 | 2.74E-02 | 7607 | 2882 | 4725 |
|  | CC | organelle | GO:0043226 | 2.62E-04 | 7190 | 2823 | 4367 |
|  | CC | intracellular organelle | GO:0043229 | 1.76E-04 | 7151 | 2800 | 4351 |
|  | CC | membrane-bounded organelle | GO:0043227 | 3.32E-06 | 6839 | 2699 | 4140 |
|  | MF | catalytic activity | GO:0003824 | 1.75E-04 | 6766 | 2300 | 4466 |
|  | CC | intracellular membrane-bounded organelle | GO:0043231 | 6.04E-07 | 6715 | 2636 | 4079 |
|  | CC | cytoplasmic part | GO:0044444 | 7.28E-13 | 5710 | 2043 | 3667 |
| S4 vs S1 | CC | cell part | GO:0044464 | 4.80E-13 | 11,139 | 4622 | 6517 |
|  | CC | intracellular part | GO:0044424 | 3.99E-10 | 9651 | 3999 | 5652 |
|  | BP | cellular process | GO:0009987 | 1.57E-06 | 9089 | 3674 | 5415 |
|  | BP | metabolic process | GO:0008152 | 4.60E-05 | 7705 | 3148 | 4557 |
|  | CC | organelle | GO:0043226 | 8.45E-10 | 7310 | 3001 | 4309 |
|  | CC | intracellular organelle | GO:0043229 | 5.75E-10 | 7269 | 2982 | 4287 |
|  | CC | membrane-bounded organelle | GO:0043227 | 8.51E-08 | 6872 | 2884 | 3988 |
|  | BP | cellular metabolic process | GO:0044237 | 1.97E-02 | 6869 | 2814 | 4055 |
|  | CC | intracellular membrane-bounded organelle | GO:0043231 | 2.92E-09 | 6760 | 2816 | 3944 |
|  | CC | cytoplasmic part | GO:0044444 | 3.82E-30 | 5911 | 2201 | 3710 |
| S4 vs S2 | CC | cell part | GO:0044464 | 2.16E-02 | 9206 | 3647 | 5559 |
|  | MF | catalytic activity | GO:0003824 | 1.10E-07 | 5779 | 2104 | 3675 |
|  | CC | cytoplasmic part | GO:0044444 | 9.07E-03 | 4629 | 1806 | 2823 |
|  | CC | membrane | GO:0016020 | 6.30E-08 | 3595 | 1324 | 2271 |
|  | BP | biosynthetic process | GO:0009058 | 1.40E-03 | 3414 | 1217 | 2197 |
|  | BP | response to stimulus | GO:0050896 | 1.67E-08 | 3300 | 1422 | 1878 |
|  | BP | organic substance biosynthetic process | GO:1901576 | 1.49E-03 | 3250 | 1140 | 2110 |
|  | BP | cellular biosynthetic process | GO:0044249 | 1.12E-03 | 3144 | 1097 | 2047 |
|  | CC | membrane part | GO:0044425 | 3.40E-02 | 3083 | 1142 | 1941 |
|  | CC | intrinsic component of membrane | GO:0031224 | 4.86E-05 | 2862 | 1046 | 1816 |
| S4 vs S3 | BP | response to stimulus | GO:0050896 | 2.58E-02 | 250 | 194 | 56 |
|  | BP | response to stress | GO:0006950 | 2.15E-02 | 181 | 139 | 42 |
|  | CC | extracellular region | GO:0005576 | 2.37E-06 | 93 | 89 | 4 |
|  | CC | cell junction | GO:0030054 | 8.61E-05 | 73 | 64 | 9 |
|  | CC | cell-cell junction | GO:0005911 | 7.96E-05 | 72 | 64 | 8 |
|  | CC | plasmodesma | GO:0009506 | 1.06E-04 | 70 | 63 | 7 |
|  | CC | endoplasmic reticulum | GO:0005783 | 7.18E-08 | 66 | 57 | 9 |
|  | CC | endoplasmic reticulum part | GO:0044432 | 1.10E-06 | 62 | 54 | 8 |
|  | CC | cell wall | GO:0005618 | 8.61E-05 | 61 | 57 | 4 |
|  | CC | external encapsulating structure | GO:0030312 | 8.61E-05 | 61 | 57 | 4 |

The top 10 enriched terms with highly significant *p*-value (≤0.05) in each comparison are represented. The values of false discovery rate, number of total, up and down-regulated were shown.

**Table S10. KEGG enrichment analysis of DEGs was compared among comparison pairs.**

| **Name** | **S2 VS S1** | | | **S3 VS S1** | | | **S3 VS S2** | | | **S4 VS S1** | | | **S4 VS S2** | | | **S4 VS S3** | | |
| --- | --- | --- | --- | --- | --- | --- | --- | --- | --- | --- | --- | --- | --- | --- | --- | --- | --- | --- |
|  | **Up** | **Down** | **Result** | **Up** | **Down** | **Result** | **Up** | **Down** | **Result** | **Up** | **Down** | **Result** | **Up** | **Down** | **Result** | **Up** | **Down** | **Result** |
| Photosynthesis | 3 | 86 | yes | 2 | 90 | yes | 13 | 38 | yes | 1 | 93 | yes | - | - | no | - | - | no |
| Photosynthesis - antenna proteins | 0 | 24 | yes | 0 | 25 | yes | - | - | no | 0 | 25 | yes | - | - | no | - | - | no |
| Ribosome | 0 | 93 | yes | 15 | 452 | yes | - | - | no | 11 | 430 | yes | - | - | no | - | - | no |
| Carbon fixation in photosynthetic organisms | 9 | 28 | yes | 21 | 64 | yes | 19 | 45 | yes | 18 | 61 | yes | 16 | 42 | yes | - | - | no |
| Porphyrin and chlorophyll metabolism | 1 | 27 | yes | - | - | no | - | - | no | 15 | 42 | yes | 19 | 26 | yes | - | - | no |
| Pentose phosphate pathway | 10 | 14 | yes | 17 | 48 | yes | 17 | 43 | yes | - | - | no | 13 | 34 | yes | - | - | no |
| Carbon metabolism | 23 | 44 | yes | 59 | 224 | yes | 61 | 210 | yes | 51 | 201 | yes | 47 | 181 | yes | - | - | no |
| Glucosinolate biosynthesis | 9 | 4 | yes | 0 | 16 | yes | - | - | no | 2 | 18 | yes | - | - | no | - | - | no |
| 2-Oxocarboxylic acid metabolism | 21 | 7 | yes | - | - | no | 22 | 67 | yes | - | - | no | 16 | 57 | yes | - | - | no |
| Sulfur metabolism | 15 | 0 | yes | 15 | 34 | yes | 9 | 44 | yes | 12 | 35 | yes | 10 | 41 | yes | - | - | no |
| Valine, leucine and isoleucine biosynthesis | 9 | 3 | yes | 38 | 21 | yes | 15 | 23 | yes | - | - | no | - | - | no | - | - | no |
| C5-Branched dibasic acid metabolism | 6 | 1 | yes | - | - | no | - | - | no | - | - | no | - | - | no | - | - | no |
| Fructose and mannose metabolism | 6 | 12 | yes | 20 | 61 | yes | 21 | 53 | yes | 21 | 54 | yes | 16 | 44 | yes | - | - | no |
| Biosynthesis of amino acids | - | - | no | 58 | 224 | yes | 54 | 230 | yes | 46 | 192 | yes | 45 | 188 | yes | - | - | no |
| Oxidative phosphorylation | - | - | no | 13 | 130 | yes | - | - | no | 11 | 107 | yes | - | - | no | - | - | no |
| DNA replication | - | - | no | 6 | 62 | yes | 7 | 55 | yes | 4 | 57 | yes | 3 | 56 | yes | - | - | no |
| Valine, leucine and isoleucine degradation | - | - | no | 16 | 25 | yes | 31 | 24 | yes | - | - | no | 24 | 17 | yes | - | - | no |
| Glycine, serine and threonine metabolism | - | - | no | 23 | 68 | yes | 21 | 67 | yes | 23 | 58 | yes | 17 | 60 | yes | - | - | no |
| Fatty acid metabolism | - | - | no | 28 | 47 | yes | 27 | 44 | yes | 32 | 40 | yes | 30 | 32 | yes | - | - | no |
| Biotin metabolism | - | - | no | 3 | 26 | yes | - | - | no | - | - | no | - | - | no | - | - | no |
| Glycolysis / Gluconeogenesis | - | - | no | 22 | 94 | yes | 23 | 84 | yes | - | - | no | 18 | 77 | yes | - | - | no |
| Biosynthesis of unsaturated fatty acids | - | - | no | 10 | 15 | yes | 9 | 13 | yes | 10 | 14 | yes | 10 | 10 | yes | - | - | no |
| Glyoxylate and dicarboxylate metabolism | - | - | no | 27 | 63 | yes | 27 | 55 | yes | 29 | 61 | yes | 27 | 44 | yes | - | - | no |
| Steroid biosynthesis | - | - | no | 14 | 32 | yes | 13 | 28 | yes | 13 | 29 | yes | 15 | 21 | yes | - | - | no |
| Amino sugar and nucleotide sugar metabolism | - | - | no | - | - | no | 20 | 144 | yes | - | - | no | 26 | 116 | yes | 19 | 1 | yes |
| Cysteine and methionine metabolism | - | - | no | - | - | no | 22 | 94 | yes | - | - | no | 21 | 81 | yes | - | - | no |
| Starch and sucrose metabolism | - | - | no | - | - | no | 29 | 121 | yes | 37 | 101 | yes | 23 | 111 | yes | - | - | no |
| Phenylalanine metabolism | - | - | no | - | - | no | 16 | 41 | yes | 28 | 27 | yes | 13 | 38 | yes | - | - | no |
| Purine metabolism | - | - | no | - | - | no | 28 | 107 | yes | - | - | no | 22 | 96 | yes | - | - | no |
| Fatty acid degradation | - | - | no | - | - | no | 24 | 21 | yes | 28 | 17 | yes | 26 | 16 | yes | - | - | no |

Result: whether the GO terms were significantly enriched by differential expressed genes. Yes: significantly enriched; no: not significant.

**Table S11. Associated genes in the glucosinolate biosynthetic pathway.**

| **Name** | **Number** | **Gene ID** | **KEGG Orthology Identifier**  **(K Number)** |
| --- | --- | --- | --- |
| leuC | 3 | BjuA015502, BjuO003799, BjuA028246 | K01703 |
| SUR1 | 6 | BjuA013283, BjuA036015, BjuB029587, BjuB048846, BjuB040479, BjuA003879 | K11819 |
| UGT74B1 | 2 | BjuA035181, BjuB017791 | K11820 |
|  |  | BjuA043506, BjuA046831, BjuB010311, BjuB030167, BjuA005541 | K11821 |
| CYP79F1 | 13 | BjuA017368, BjuA022066, BjuB021730, BjuB027353, BjuB027148, BjuA001684, BjuA018402, BjuB017208, BjuA002613, BjuB024228, BjuA039949, BjuB038533, BjuO008644 | K12154 |
| CYP83A1 | 5 | BjuA015480, BjuA017367, BjuB027146, BjuA028230, BjuB016796 | K12156 |
| MAM1 | 14 | BjuA007757, BjuA042222, BjuA045597, juA042223, BjuB037742, BjuA007825, BjuO012825, BjuA042220, BjuB032394, BjuB029278, BjuA044305, BjuA022211, BjuA016955, BjuA045596 | K15741 |
| MAM3 | 1 | BjuB041431 | K15742 |
| CYP83B1 | 2 | BjuB016796, BjuA028230 | K11818 |
| BCAT4 | 3 | BjuB025120, BjuB044984, BjuA012656 | K21346 |
| CYP79F2 | 2 | BjuA022066, BjuB021730 | K12155 |
| CYP79B2 | 5 | BjuA002613, BjuB038533, BjuA014292, BjuO008644, BjuB024228 | K11812 |
| CYP79B3 | 4 | BjuB024228, BjuA002613, BjuB038533, BjuO008644 | K11813 |
| CYP79A2 | 1 | BjuA039949 | K12153 |

**Table S12.** Differentially expressed genes in the glucosinolate biosynthetic pathway.

| **Comparison Pair** | **Total**  **Number** | **Up Regulated Gene** | **Down Regulated Gene** |
| --- | --- | --- | --- |
| S2 vs S1 | 13 | BjuA015502, BjuA022066, BjuA035181, BjuA036015, BjuA043506, BjuB017791, BjuB021730, BjuB029587, BjuB030167, BjuA007757, BjuA013283, BjuB041431, BjuB048846 | BjuA007757, BjuA013283, BjuB041431, BjuB048846 |
| S3 vs S1 | 16 | -- | BjuA007757, BjuA013283, BjuA015480, BjuA015502, BjuA017368, BjuA022066, BjuA035181, BjuA036015, BjuA042222, BjuA045597, BjuB017791, BjuB021730, BjuB027146, BjuB0295879, BjuB041431, BjuB048846 |
| S3 vs S2 | 19 | BjuB027353 | BjuA015480, BjuA015502, BjuA017367, BjuA017368, BjuA022066, BjuA035181, BjuA036015, BjuA042222, BjuA043506, BjuA045597, BjuB010311, BjuB017791, BjuB021730, BjuB027146, BjuB029587, BjuB030167, BjuB041431, BjuO003799 |
| S4 vs S1 | 20 | BjuA015502, BjuA046831 | BjuA007757, BjuA013283, BjuA015480, BjuA017368, BjuA022066, BjuA035181, BjuA036015, BjuA042222, BjuA045597, BjuB016796, BjuB0177910, BjuB021730, BjuB027146, BjuB029587, BjuB030167, BjuB041431, BjuB048846, BjuO003799 |
| S4 vs S2 | 17 | BjuA046831 | juA015480, BjuA017367, BjuA017368, BjuA022066, BjuA035181, BjuA036015, BjuA042222, BjuA0435061, BjuA045597, BjuB017791, BjuB021730, BjuB027146, BjuB029587, BjuB030167, BjuB041431, BjuO003799 |
| S4 vs S3 | 1 | BjuA015502 | -- |

**Table S13.** The contents and distribution of glucosinolates in different stages in stem mustard.

| Gulcosinolates  Composition | **Content (μmol/g DW)** | | | |
| --- | --- | --- | --- | --- |
|  | **Stage 1** | **Stage 2** | **Stage 3** | **Stage 4** |
| Sinigrin | 14.53 ± 1.20 | 9.14 ± 1.07 | 2.23 ± 1.06 | 0.17 ± 0.10 |
| Glucoalyssin | 0.09 ± 0.03 | 0.08 ± 0.01 | 0.05 ± 0.01 | 0.06 ± 0.03 |
| Gluconapin | 0.32 ± 0.03 | 0.16 ± 0.02 | 0.05 ± 0.01 | 0.02 ± 0.01 |
| Total aliphatic | 14.94 ± 1.25 | 9.37 ± 1.10 | 2.33 ± 1.07 | 0.25 ± 0.14 |
| 4-Hydroxy glucobrassicin | 0.13 ± 0.03 | 0.07 ± 0.00 | 0.02 ± 0.01 | 0.01 ± 0.00 |
| Glucobrassicin | 0.01 ± 0.00 | 0.01 ± 0.00 | 0.002 ± 0.00 | 0.002 ± 0.00 |
| 4-Methoxy glucobrassicin | 0.04 ± 0.00 | 0.03 ± 0.00 | 0.07 ± 0.03 | 0.04 ± 0.01 |
| Neoglucobrassicin | 0.06 ± 0.01 | 0.02 ± 0.00 | 0.04 ± 0.02 | 0.01 ± 0.00 |
| Total indole | 0.25 ± 0.04 | 0.13 ± 0.00 | 0.13 ± 0.05 | 0.05 ± 0.02 |
| Gluconasturtiin | 0.54 ± 0.07 | 0.43 ± 0.05 | 0.21 ± 0.09 | 0.27 ± 0.12 |
| Total aromatic | 0.54 ± 0.07 | 0.43 ± 0.05 | 0.21 ± 0.09 | 0.27 ± 0.12 |
| Total gulcosinolates | 14.94 ± 1.25 | 9.37 ± 1.10 | 2.33 ± 1.07 | 0.25 ± 0.14 |

**Table S14.** The correlation between the glucosinolates compounds and development stage was expressed.

|  |  | **Aliphatic Glucosinolate** | | | **Indole Glucosinolate** | | | | **Aromatic Glucosinolate** |
| --- | --- | --- | --- | --- | --- | --- | --- | --- | --- |
|  | **Developm-ent Stage** | **Sinigrin** | **Glucoalyssin** | **Gluconapin** | **4-Hydroxy Glucobrassicin** | **Glucobrassicin** | **4-Methoxy**  **Glucobrassicin** | **Neoglucobrassicin** | **Gluconasturtiin** |
| Development stage | 1 |  |  |  |  |  |  |  |  |
| Sinigrin  Glucoalyssin  Gluconapin  4-Hydroxy glucobrassicin  Glucobrassicin  4-Methoxyglucobrassicin  Neoglucobrassicin  Gluconasturtiin | -0.976**  -0.581  -0.957**  -0.939**  -0.892**  0.156  -0.789*  -0.823** | 0.666  0.986**  0.979**  0.918**  -0.240  0.758*  0.910** | 0.662  0.740*  0.697*  -0.108  0.523  0.758* | 0.981**  0.927**  -0.223  0.803**  0.905** | 0.899**  -0.239  0.778*  0.904** | -0.263  0.749*  0.816** | 0.339  0.372 | 0.621 | 1 |

* means significant differences at 0.05 level; ** means significant differences at 0.01 level.

**Table S15. Sequences of primers used in qPCR.**

| **Gene ID** | **Forward Primer (5'→3')** | **Reverse Primer (5'→3')** |
| --- | --- | --- |
| *BjuO003799* | AGGTGTGGATGGATGTGTATGC | GCGGTGTAAGGAGAAGCCAAG |
| *BjuA036015* | TCTCACGACCATCTCCACAAGG | GCGATCCAGCCAATCTTCCATC |
| *BjuB017791* | ATCGCCACCACCACCTACAC | ACGGACAAGATACGGAGCAGAG |
| *BjuA046831* | ACGAGAATGTGAGTGCTGATCC | CATCCATACGAGCTGCCATGTC |
| *BjuB021730* | GAAGGTTAGGACCAGCGGAGAA | CGGATTGTTGTAACTGCGGACT |
| *BjuA015480* | CCGCAGTTCTCCTCTTCTTCCT | ATCATCGTCCTAGCCTCCTCCT |
| *BjuA045597* | GAAGATGGCGGCAGGTCTGA | GGCGATTGTGTTGGCGGTAG |
| *BjuB041431* | GAGAGTGGCATTCACCAGGATG | CGTTAAGGTCAGCGTCCGTAAT |
| *BjuB016796* | ATGGTAGCGGTCACAGCCTTC | AGGAGAGGACGAGCGGTGAA |
| *UBC* | ATCGTCTGTGACAACGGTAC | GATACGGAGCTCGTTGTAGA |
